# Accuracy of intraoral scanning versus conventional impressions for partial edentulous patients with maxillary defects

**DOI:** 10.1038/s41598-023-44033-6

**Published:** 2023-10-05

**Authors:** Rongkai Cao, Shilei Zhang, Lishan Li, Piaopiao Qiu, Hui Xu, Yujie Cao

**Affiliations:** 1 Department of Stomatology, The First Affiliated Hospital, Fujian Medical University, No. 20, Chazhong Rd, Fuzhou, 350005 Fujian China; 2 Department of Stomatology, National Regional Medical Center, Binhai Campus of the First Affiliated Hospital, Fujian Medical University, Fuzhou, 350212 China; 3 General Dentistry, Stomatological Hospital of Xiamen Medical College, Xiamen, 361008 China; 4 School & Hospital of Stomatology, Shanghai Engineering Research Center of Tooth Restoration and Regeneration, Tongji University, Shanghai, 200072 China

**Keywords:** Occlusion, Prosthetic dentistry

## Abstract

This study aimed to evaluate the accuracy of digital dental impressions obtained by intraoral scanning (IOS) for partial edentulous patients with maxillary defects by comparing them with conventional impression techniques. Ten subjects underwent an experimental procedure where three ceramic blocks were affixed to the healthy palate mucosa. Digital dental impressions were captured using IOS and subsequently imported into software. Conventional impressions obtained by silicone rubber were also taken and scanned. Linear distance and best-fit algorithm measurements were performed using conventional impression techniques as the reference. Twenty impressions were analyzed, which included 30 pairs of linear distances and 10 best-fit algorithm measurements. Regarding linear distance, paired two-sample t-test demonstrated no significant differences between IOS and model scanning in groups A and C, whereas significant differences were found in group B (P < 0.05). Additionally, ANOVA revealed significant differences among the groups (P < 0.05). No significant differences were found for the best-fit algorithm measurement of the dentition. IOS can provide accurate impressions for partial edentulous patients with maxillary defects and its accuracy was found to be comparable with conventional impression techniques. A functional impression may be needed to ensure accurate reproduction of soft and hard tissues in defect or flap areas.

## Introduction

Making dental impressions, which is usually considered to be the first step for dental procedures, is necessary in most clinical settings^1^. In clinical scenarios, conventional dental impressions obtained by alginate or silicone rubber were applied to measure the occlusal relationship and dentition. However, this method is associated with several drawbacks, including the significant time and cost involved in model fabrication, impression-taking, and model storage^2^. Furthermore, research has shown that conventional dental impressions are prone to inaccuracies due to factors such as potential distortion and expansion of gypsum casts, as well as changes in shape over time when impressions are sent to dental laboratories^3–5^. Digital dental impressions, which have been gradually applied in clinical practice, have emerged as a promising solution to the limitations of traditional dental impressions. Compared to conventional methods, digital dental impressions offer several advantages, such as real-time imaging and evaluation, less requirement for materials, and improved cost-effectiveness and communication^6^. Studies have shown that their accuracy can be acceptable in clinical settings compared with conventional ones^7–10^.

In general, digital dental impressions can be acquired either by scanning the conventional impressions or by IOS with an intraoral scanner. The accuracy of dental extraoral laboratory scanners has been evaluated in previous studies^11,12^. Data acquired using IOS is comparable to that obtained using conventional methods for single crowns and partial fixed prostheses^13,14^. Nevertheless, researchers have reported that extraoral scanners demonstrated higher accuracy measurements for the cross-arch measurement^15^. In addition, studies have found that prosthesis fabricated from model scanning has lower average discrepancies than those produced using IOS^16^.

Maxillary tumors can result in significant loss of hard and soft tissues, leading to maxillary defects and decreased quality of life for patients. Therefore, treatment of the maxillary defects should focus on minimizing potential problems and preserving quality of life. The fabrication of a maxillary obturator can help to address these issues, and reports have shown that patients experience improved satisfaction with the use of a maxillary obturator^17,18^. For partial edentulous patients with maxillary defects, conventional dental impressions using silicone rubber or alginate are still widely used in clinical practice due to their acceptable accuracy and feasibility^19,20^. Nonetheless, these patients may experience difficulties with mouth opening due to scar contracture or temporomandibular joint disease, which can impact the accuracy and feasibility of conventional dental impressions. Moreover, defective tissues of the upper palate and the penetration between the oral cavity and nasal cavity may cause materials to enter the nasopharynx cavity, bringing pain and discomfort to patients. Accordingly, making dental impressions for this specific clinical population, partial edentulous patients with maxillary defects, could be an enormous challenge for dentists during the clinical practice of dentistry.

With the rapid advancement of digital technology in dentistry, practitioners can now obtain 3D scans of hard and soft tissues and occlusion relationships using intraoral scanners. This method eliminates the drawbacks of conventional dental impressions and enables practitioners to digitally evaluate and design the maxillary obturator. Compared to conventional impression techniques, the use of IOS simplifies the workflow and avoids time consumption and potential deviations^21^. Trueness, which describes the congruence between a prototype STL dataset and a control STL dataset, is considered a crucial factor in determining the ability of IOS to capture dental impressions with high quality^22,23^.

The accuracy of IOS has been reported to decrease when scanning larger areas compared with a short span^24,25^. Therefore, the aim of the present study was to assess the accuracy of digital dental impressions obtained from IOS for partial edentulous patients with maxillary defects by comparing the linear distance and best-fit algorithm measurements with those obtained from conventional impression techniques in a quantitative manner.

## Materials and methods

The present study was approved by the Institutional Review Board of the First Affiliated Hospital of Fujian Medical University, Fujian, PR CHINA (No. MRCTA, ECFAH of FMU [2020] 430). Written informed consents were obtained from ten partial edentulous patients with maxillary defects in full accordance with the ethical principles of the World Medical Association Declaration of Helsinki (version 2008). The following inclusion criteria were applied: (1) age ≥ 18 years; (2) mild to moderate limitation of mouth opening; (3) inability or refusal to undergo implant surgery due to physical conditions, but required prosthetic restoration. The excursion criteria were (1) severe mouth opening limitation; (2) tooth mobility > I.

### Placement of ceramic blocks

Before the experiment, the dentition and mucosa of the subjects were isolated and dried using an air syringe, and cottons. To protect the mucosa of the defect areas, vaseline gauze was used. Three customized zirconia ceramic blocks (6 mm × 6 mm × 3 mm) were attached to the healthy palate mucosa of residual dentition and the defect area using medical tissue glue (Medical Tissue Glue, B. Braun Corp, China). The ceramic block at the anterior position of the residual dentition was marked as A block, the block placed near the defect area or flap area was labeled as B block, and the ceramic block at the posterior of the residual dentition was marked as C block.

### Intraoral scanning

**Figure 1:**
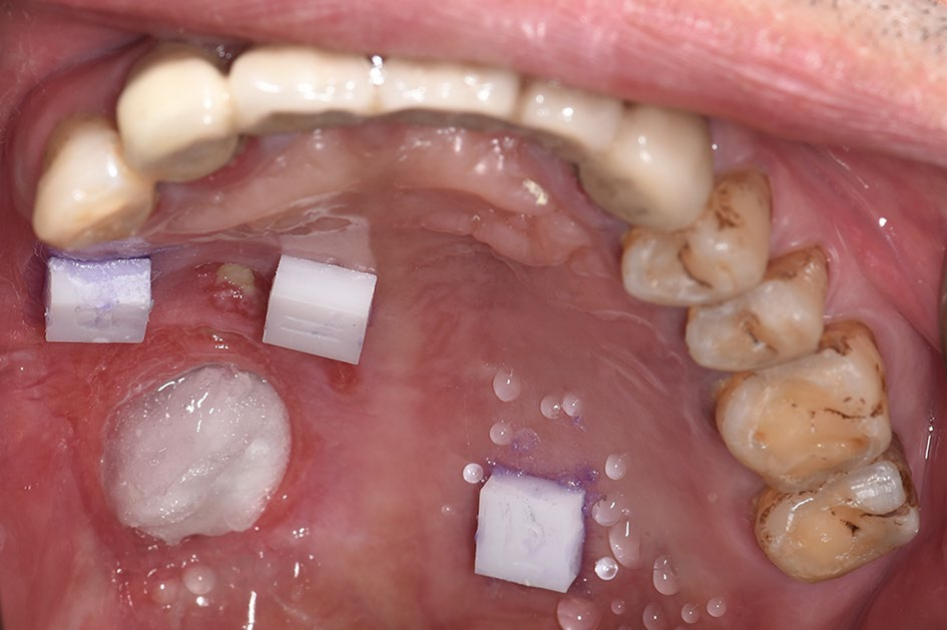
Images of the maxillary of subjects after ceramic blocks were pasted.

**Figure 2:**
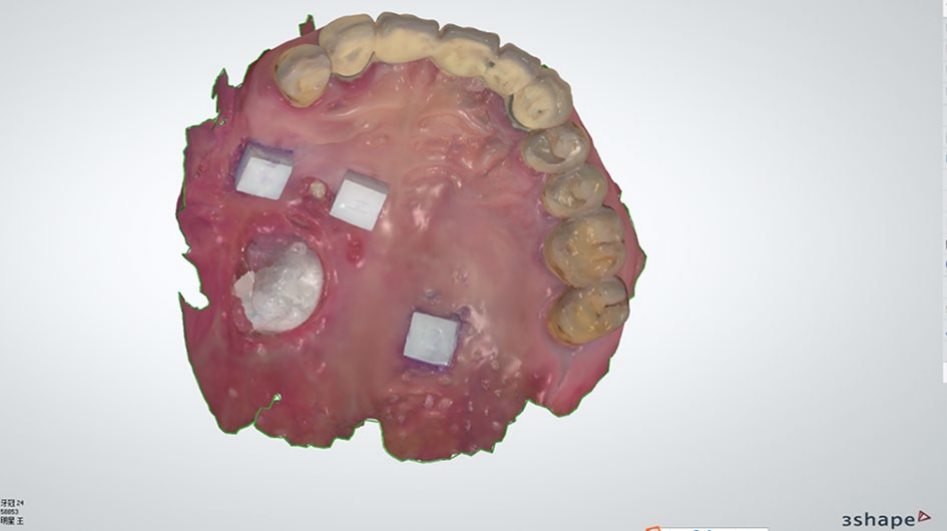
Digital dental impressions obtained by intraoral scanning.

Each patient received both conventional impression-taking and IOS. The digital dental impression of IOS was before the conventional impression techniques procedure. Digital dental impressions were obtained using an intraoral scanner (TRIOS color, 3Shape, Denmark) by an experienced dentist. Starting from the occlusal-palatal side of posterior teeth in the first quadrant, the intraoral scanner was turned to the buccal side of the teeth in the second quadrant and then returned to the first quadrant. The mucosa of each patient was also scanned, and the scan data was saved and exported in the STL format (Figs. 1, 2).

### Conventional impression techniques

**Figure 3:**
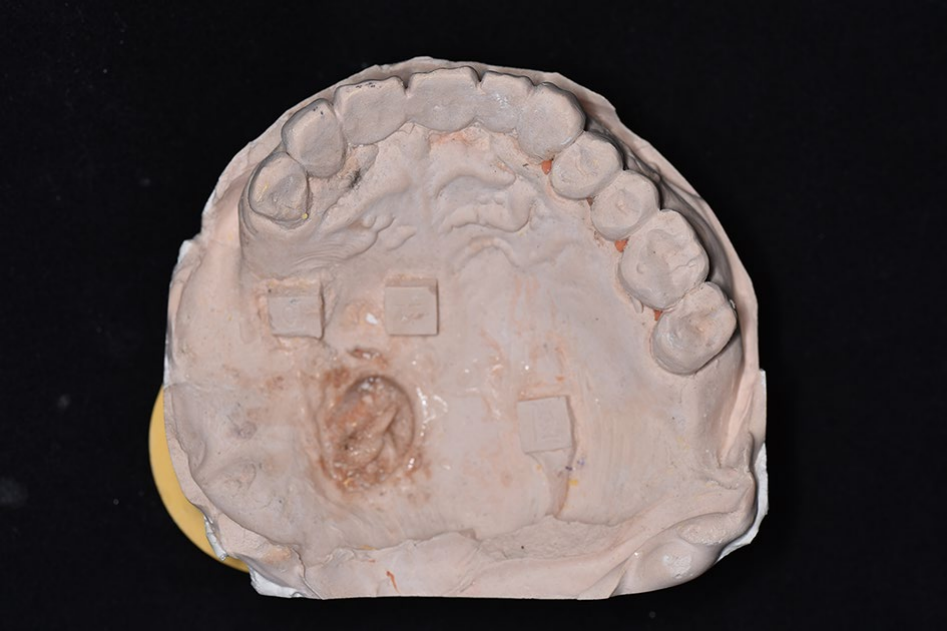
Maxillary plaster dental impressions obtained by silicone rubber using conventional impression techniques.

**Figure 4:**
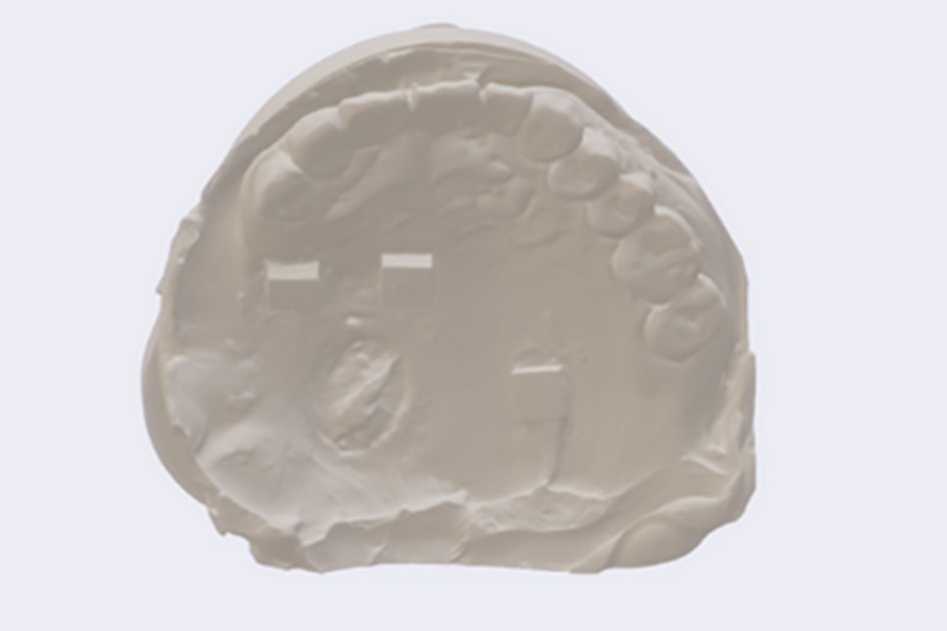
Digital dental impressions obtained by model scanning.

For each participant, maxillary silicone rubber impressions (Meijiayin elastomer impression material type III, HuGe, China) were taken using full-arch metal stock impression trays in accordance with the manufacturer’s instructions under the same conditions. The dental impressions were then poured with dental stone (Die-stone, Heraeus Kulzer, USA) by the same experienced dentist. After 40 min, impression trays were removed from the stone models. The stone models were then scanned by using a desktop scanner (E2 lab scanner, 3shape, Denmark) and saved in STL format (Figs. 3, 4).

### Measurement procedure

**Figure 5:**
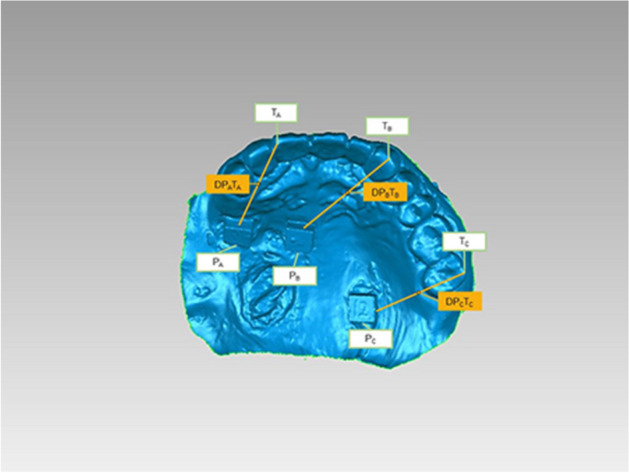
Linear distance between ceramic block and corresponding tooth.

**Figure 6:**
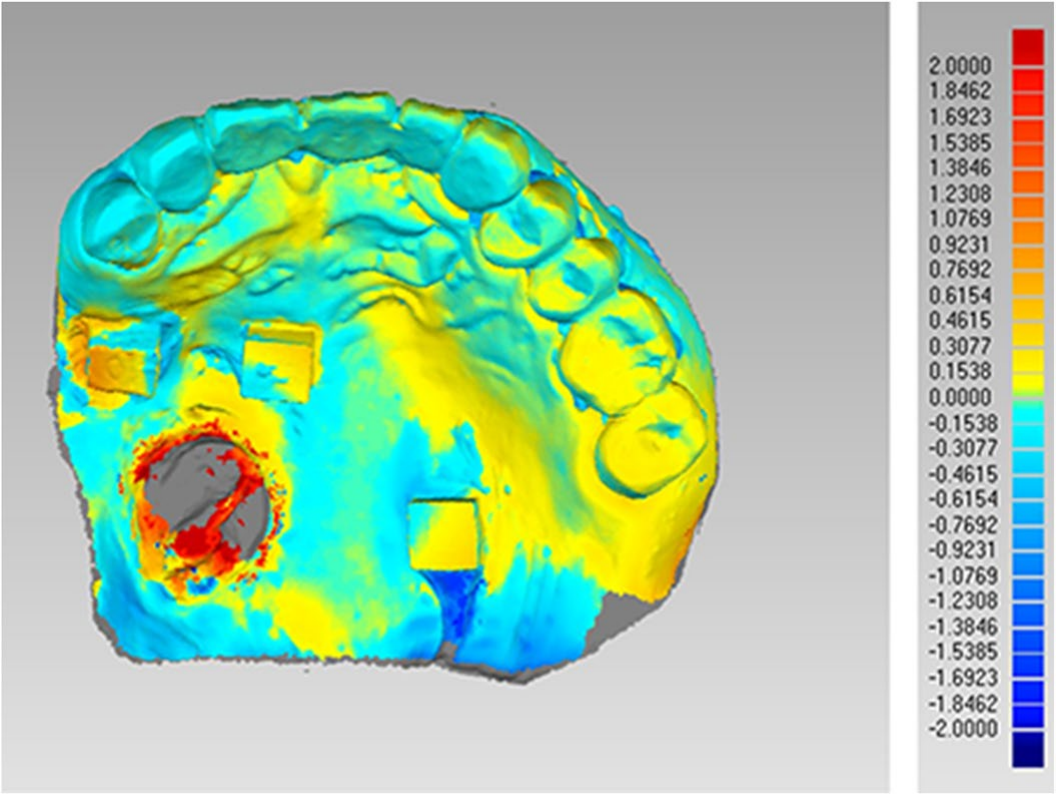
Models obtained by intraoral scanning and conventional impressions superimposed with best-fit algorithm.

**Figure 7:**
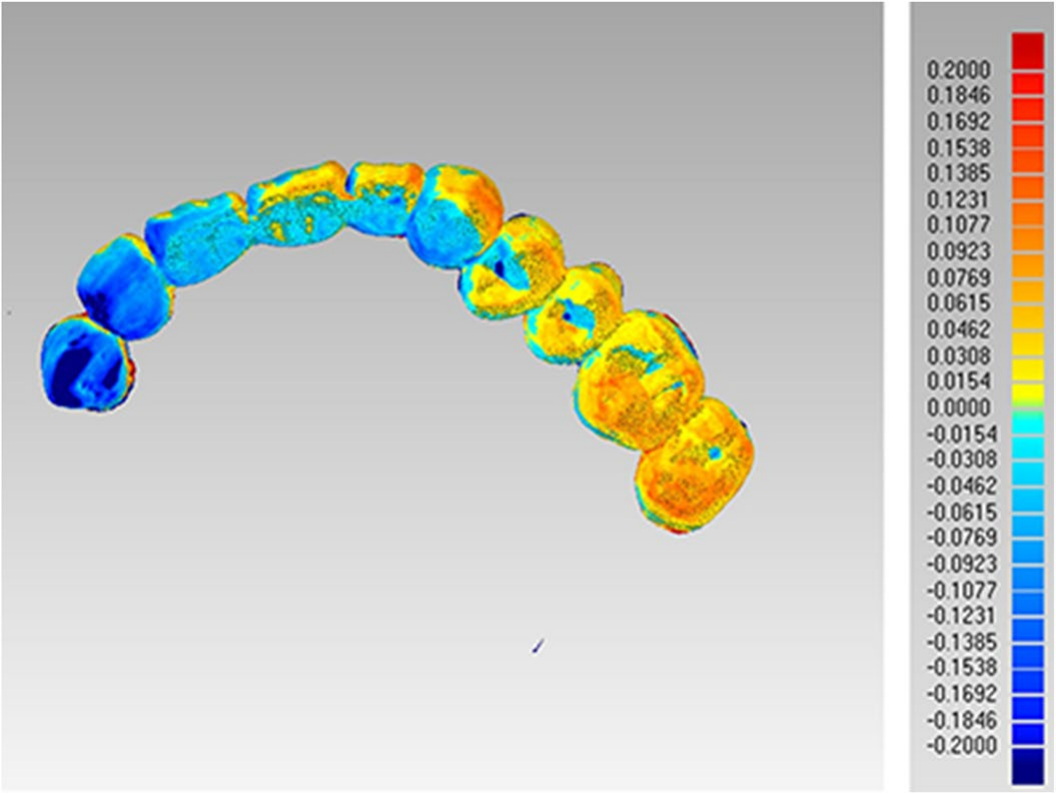
Best-fit algorithm measurement of maxillary dental arch.

The scan files of IOS and model scanning were converted to three-dimensional (3D) images by a reverse engineering software program (Geomagic Wrap, Raindrop Geomagi, USA) for matching and data measurement. Two measurements were performed for each subject, the linear distance between each ceramic block and the corresponding tooth point and the best-fit algorithm measurement. The linear distance was defined as the distance between the central point of each ceramic block and the corresponding point of the residual teeth (Fig. 5). Three linear distances between ceramic blocks A, B, and C were measured. The measurements of linear distance between two ceramic blocks were the Euclidean distance calculated based on the data obtained in the software. The best-fit alignment measurement utilizes an iterative closest point algorithm to superimpose two meshes and minimize the difference between the two clouds of points and iteratively revise the transformation needed to minimize an error metric.^26^ The entire dataset obtained by IOS and conventional impression techniques was aligned to generate deviation maps containing information including median and average deviation and analyzed using the best-fit alignment method on the software (Fig. 6). Moreover, the dentition of each group of models was trimmed along the tooth neckline for further statistical analysis in the software (Fig. 7). The accuracy of the entire dataset best-fit algorithms alignment method has been demonstrated in the literature^27^.

### Statistical analysis

Data analysis was performed using SPSS 26.0 software (IBM SPSS Statistics, IBM, USA). Statistical analysis was performed using the Shapiro–Wilk test for normal distribution and Levene’s test for homogeneity of variance. A paired two-sample t-test was applied to assess the differences in the linear distance between the ceramic block and the corresponding point of the residual teeth in each group. The differences in linear distance between IOS and conventional impression techniques in the three blocks of each patient were recorded. Subsequently, the differences in three groups were compared using one way ANOVA test to evaluate the accuracy of digital impressions, and the least significant difference test was applied for further comparison in pairs. The best-fit algorithm measurement of the dentition was assessed using the Kolmogorov–Smirnoff test. In all statistical analyses, the level of significance was set to α = 0.05.

### Ethics approval

The study protocol was approved by the Institutional Review Board of the First Affiliated Hospital of Fujian Medical University, Fujian, PR CHINA (Approval Number MRCTA, ECFAH of FMU [2020]430).

### Informed consent

Interventions were undertaken with the understanding and written informed consent of each subject. The patients were treated in full accordance with ethical principles, including the World Medical Association Declaration of Helsinki (version 2008).

## Results

Twenty digital dental impressions of ten eligible subjects were analyzed, which included 30 pairs of linear distances (IOS and model scanning) and 10 best-fit algorithm measurements of the dentition.

### Linear distance

Overall, significant differences were found between IOS and conventional impression techniques in terms of the linear distance. Regarding the linear distance within each group, the paired t-test revealed no difference between IOS and conventional impression techniques in ceramic blocks of group A and group C, while significant differences were observed for group B (P < 0.05), indicating the deviations in digital dental impressions obtained by IOS compared to the conventional impression techniques in this area (Table 1). Moreover, the results of ANOVA showed that there were differences among groups (P < 0.05), indicating that the position of the ceramic block had an impact on the accuracy of digital dental impressions (Table 2). After using the least significant difference test, the results showed that there were significant differences in the linear distance of IOS and conventional impression techniques between group B and group C (P < 0.05), while no differences were found when comparing to group A, which demonstrated that ceramic block A had a relatively stable position with less displacement during the process of making dental impressions (Table 3).

**Table 1:** Differences of linear distance between conventional impressions and intraoral scanning.

Group	n	Mean ± SD	t	P
A	10	−0.038 ± 0.238	−0.502	0.628
B	10	0.327 ± 0.350	−2.985	0.015
C	10	−0.109 ± 0.412	−0.674	0.517
Total	30	−0.158 ± 0.389	−2.198	0.034

**Table 2:** Results of ANOVA for linear distance among groups.

Group	n	Mean ± SD	F	P
A	10	−0.038 ± 0.238	3.272	0.049
B	10	0.327 ± 0.350		
C	10	−0.109 ± 0.412

**Table 3:** Results of least significant difference test in terms of ceramic position among groups.

(I) group	(J) group	Xi − Xj	P
A	B	−0.288	0.102
	C	0.147	0.397
B	A	0.288	0.102
	C	0.435	0.017
C	A	−0.147	0.397
	B	−0.435	0.017

### Best-fit algorithm

The results from the best-fit algorithm measurement of the two groups showed that errors were basically concentrated in the maxillary defect areas (> 2 mm) (Fig. 6). The results of the Kolmogorov-Smirnoff test for residual dentition showed that there was no significant difference between IOS and conventional impression techniques (P = 0.18) (Table 4) (Fig. 7), indicating the dentition of digital dental impressions obtained by IOS can be acceptable in clinical settings.

**Table 4:** Results of best-fit algorithm measurement of residual dentition in terms of accuracy between conventional impressions and intraoral scanning.

n	Median	Z	P
10	0.200	1.329	0.184

## Discussion

The present study was performed to evaluate the accuracy of IOS for partial edentulous patients with maxillary defects. In the present comparative clinical study, the feasibility of making dental impressions for partial edentulous patients with maxillary defects using IOS was illustrated and assessed, which provided references for clinical restorative treatment. To our knowledge, this is one of the first investigations which focus on digital dental impressions obtained by an intraoral scanner for this specific clinical population.

The accuracy of dental impressions is a critical step in ensuring predictable treatments and long-lasting restorations^28^. The accuracy of digital dental impressions has been demonstrated in several studies^7–10^. In clinical scenarios, three methods are currently used to obtain digital dental impressions, including IOS, extraoral model scanning, and cone beam compute tomography (CBCT). Trueness and precision are two components of accuracy, and this study assessed only the trueness of IOS as the evaluation of precision would require comparison between different datasets obtained using the same method. However, due to the discomfort associated with traditional dental impressions for partial edentulous patients with maxillary defects, this study avoided taking multiple impressions for the same patient. The accuracy of the model scanning has been verified and the silicone rubber impression material demonstrated excellent dimensional stability, detail reproducibility, and gypsum compatibility^29^. In addition, the comparison between the IOS and extraoral model scanning of conventional impression techniques has been applied in many different scenarios in previous studies^30–33^. On the other hand, CBCT was excluded from this study due to radiation and accuracy concerns.

Although widely used in the clinical practice of dentistry, conventional impression techniques obtained with silicone material may introduce inaccuracies due to the contraction of the impression material and expansion of the stone. In addition, this method is associated with several drawbacks, including the significant time and cost involved in model fabrication, impression-taking, and model storage^2^. With the rapid advancement of digital technology, the digital workflow with the help of IOS avoids the time consumption and potential deviations of impression preparation and gypsum pouring using conventional dental impression techniques. In addition, it is convenient for clinicians to re-scan and make up for the faults that occurred in the procedures of making impressions, while it might need to remake a new one if there is something wrong with conventional impression techniques. However, the accuracy of digital impressions obtained by IOS in the soft tissue of the maxillary remains controversial in the literature^33^. Nevertheless, when utilized appropriately, IOS can provide clinicians with valuable feedback, particularly regarding impression quality and the overall shape and geometry of preparations^34^.

To assess the accuracy of IOS, various measurement schemes have been adopted, mainly including linear distance and best-fit algorithm measurement^35–37^. In this study, best-fit algorithm measurement was selected for analysis due to its ability to reduce error compared to linear distance by using larger data sets and better detecting impression deviations. Moreover, this approach can visualize superposition results with the help of distance maps and evaluate the differences more intuitively. However, some research has suggested that the best-fit algorithm is not widely applicable due to its potential to automatically correct large differences in one region and cause registration errors in other areas, especially in large areas or soft tissues^38^. In addition, the different alignment protocols and versions among software may influence the results of best-fit algorithm measurement^39^. Linear distance was also used to measure the accuracy of complete dentition obtained by IOS in the literature^10^. Therefore, linear distance and best-fit algorithm measurement were used in this study to improve the reliability.

Previous studies have shown that applying markers on the mucosa can aid in the acquisition of digital dental impressions^40^. Several kinds of artificial markers have been applied to assess the evaluation of the accuracy of IOS for digital dental impressions. For example, a previous study fixed 4 metal spheres with composite aid to the mandibular teeth^10^. Other potential approaches include drawing irregular shapes and lines with a mixture of pressure-indicating paste and zinc oxide-eugenol cement^41^. In addition, Fang et al. injected flowable composite resin to six different sites on the hard palate and light-polymerized the resin to facilitate the application of IOS to make digital impressions of edentulous jaws with a broad palate^42^. In this study, to analyze the influence of mobility of soft tissue and tooth morphology on the accuracy of IOS, three customized zirconia ceramic blocks of 6 mm × 6 mm × 3 mm were placed at the anterior teeth, posterior teeth, and near the defect or flap area according to specifications. After cutting and sintering, the edges of the ceramic blocks were polished to ensure an error of less than 0.1 mm. Due to the lack of structure that needs to be recognized by an intraoral scanner, bonding ceramic blocks instead of resin or metal spheres can assist in matching generated images and reducing operation time during IOS by providing structure to be recognized by the intraoral scanner. On the other hand, the center of the ceramic block can be used for subsequent measurement and data analysis.

Paired t-test results showed no difference between group A and group C, while significant differences were observed in group B. This may be due to the ceramic block in group B being close to a defect or flap area with large mobility, resulting in deviations in digital dental impressions obtained by IOS compared to conventional impression techniques. In addition, the least significant difference test showed differences between group B and group C, while no differences were found when comparing with group A. This may be due to the ceramic block in group A being located on the palatal mucosa near the anterior teeth, which had a relatively stable position with less displacement during the process of making dental impressions. In contrast, the ceramic blocks in group C located near the hard-soft palate junction area may change position with the movement of the mucosa during operation. The inconspicuous change superimposed with displacement of the ceramic block in group B led to the differences in the accuracy of IOS between group B and group C. The results of the Kolmogorov–Smirnoff test for residual dentition indicated the digital dental impressions obtained by IOS can be acceptable in clinical settings. Therefore, the accuracy of digital dental impressions obtained by IOS can meet requirements for the fabrication of scaffolds, retainers and small connectors. However, results from best-fit algorithm measurement showed that errors were basically concentrated in the maxillary defect areas (> 2 mm) (Fig. 6). Therefore, when using digital dental impressions for a mental scaffold obturator in clinical practice, a functional impression should be combined to accurately reproduce the shape of soft and hard tissues in defect areas under functional status after scaffold and tooth setting are completed on the digital models to complete the fabrication of obturator.

This study has several limitations. First of all, using the model scanning of rubber dental impressions obtained by silicone material may introduce inaccuracies due to the contraction of the impression material, expansion of the stone, and resolution limitations of the non-industrial model scanner. In addition, due to the discomfort of partial edentulous patients with maxillary defects when making conventional dental impressions, the precision of IOS was not evaluated in this study. Finally, it must be taken into account that the sample size was relatively small, and future clinical research with a larger sample size should better analyze the potential distortions between IOS and conventional impressions and assess the feasibility and efficacy of IOS in clinical scenarios.

## Conclusions

Based on the data analyzed in this study, the following conclusions were drawn:

1. IOS can provide accurate digital dental impressions for partial edentulous patients with maxillary defects. The accuracy of IOS was found to be comparable to that of conventional impression techniques.
2. The digital dental impressions obtained by IOS can be acceptable for the fabrication of scaffolds, retainers, and small connectors in clinical settings.
3. To ensure accurate reproduction of soft and hard tissues in defect or flap areas, a functional impression may be needed in addition to the digital dental impression obtained by IOS.

## Data Availability

The data used or analyzed during the current study are available from the corresponding author upon reasonable request.
